# Perceptual stimuli with novel bindings interfere with visual working memory

**DOI:** 10.3758/s13414-021-02359-1

**Published:** 2021-09-03

**Authors:** Peter Shepherdson

**Affiliations:** 1 University of Akureyri, Norðurslóð 2, 600 Akureyri, Iceland; 2 University of Zurich, Zürich, Switzerland

**Keywords:** Visual working memory, Interference, Change detection, Recall

## Abstract

What influences the extent to which perceptual information interferes with the contents of visual working memory? In two experiments using a combination of change detection and continuous reproduction tasks, I show that binding novelty is a key factor in producing interference. In Experiment 2, participants viewed arrays of colored circles, then completed consecutive change detection and recall tests of their memory for stochastically independent items from the same array. When the probe used in the change detection test was novel (i.e., required a “change” response), subsequent recall performance was worse than in trials with matching (i.e., “no change”) probes, irrespective of whether or not the same item was tested in both phases. In Experiment 2, participants viewed arrays of oriented arrows, then completed a change detection (requiring memory) or direction judgement (not requiring memory) test, followed by recalling a stochastically independent item. Again, novel probes in the first phase led to worse recall, irrespective of whether the initial task required memory. This effect held whether the probe was wholly novel (i.e., a new feature presented at any location) or simply involved a novel binding (i.e., an old feature presented at a new location). These findings highlight the role of novelty in visual interference, consistent with the assumptions of computational models of WM, and suggest that new bindings of old information are sufficient to produce such interference.

**Supplementary Information:**

The online version contains supplementary material available at 10.3758/s13414-021-02359-1.

One of the key characteristics of working memory (WM) is its flexibility (e.g., Gilchrist & Cowan, 2014; Oberauer, Souza, Druey, & Gade, 2013; Shipstead, Harrison, & Engle, 2016). As a severely capacity-limited system, this flexibility is vital in ensuring its usefulness. Without the ability to rapidly and adaptively modify the contents of WM, the utility of the system would likely be as limited as its capacity.

The advantages obtained from the flexibility of WM are not without accompanying disadvantages. Thus, information in WM is subject to loss (Zhang & Luck, 2009), replacement (Nosofsky & Donkin, 2016), and interference from other information (Oberauer & Lin, 2017). Controlling the influence of irrelevant information on WM is important to the effective functioning of the system, to the extent that the facility with which people can prevent irrelevant information from being encoded or retained in WM has been proposed as an explanation for individual differences in WM capacity (e.g., Vogel, McCollough, & Machizawa, 2005). However, the characteristics of distracting information itself also affect the extent of WM disruption (e.g., Allen, Castellà, Ueno, Hitch, & Baddeley, 2014; Fiacconi, Cali, Lupiáñez, & Milliken, 2020; Sligte, Scholte, & Lamme, 2008).

In the experiments described here, I examined how one particular characteristic of distracting perceptual information—namely, its novelty—affects the extent to which it undermines people’s ability to retain existing task-relevant information in memory. Next, I outline the background to this issue, then describe the means I used to investigate it.

## Novelty and distraction in working memory

The novelty of a stimulus has a number of implications for the manner in which it is processed (for review, see Schomaker & Meeter, 2015). For instance, stimulus novelty is detected even when stimuli are not attended (Tarbi, Sun, Holcomb, & Daffner, 2011), and novel stimuli better capture attention (Escera, Alho, Winkler, & Räätänen, 1998) and are encoded more effectively into episodic long-term memory compared to non-novel stimuli (Tulving & Kroll, 1995).

Novelty often has beneficial effects when it is a feature of task-relevant stimuli; yet when distracting stimuli are novel, this can be detrimental to performance. This idea is captured in a prominent computational model of verbal working memory, SOB, and its offshoots (e.g., Farrell & Lewandowsky, 2002; Oberauer, Lewandowsky, Farrell, Jarrold, & Greaves, 2012; Lewandowsky & Oberauer, 2015; Oberauer & Lewandowsky, 2014). SOB includes the assumption that encoding into WM is novelty-dependent, with more rapid encoding of stimuli that are more novel. Importantly, novelty is calculated in the model on the basis of a comparison between a newly presented stimulus and the existing bindings in memory between stimulus contents (e.g., a specific word) and the contexts in which they were presented (e.g., serial order). For instance, if the ordered word list *stick, tile, key, bird* were followed by a second presentation of the word “stick”, this final word’s novelty value (and thus its encoding strength) would be greater than if the preceding list had been ordered *bird, key, tile, stick*. In both cases, the content (“stick”) is already in memory, but in the former instance, the mismatch between the context (here, serial position) of the new instance and the old one is greater (position 5 vs. 1 in the first list, compared to position 5 vs. 4 in the second).

In SOB, novelty-dependent stimulus encoding is used to account for the greater effect of unique distractors on serial recall from WM relative to repeated distractors. For example, in a complex span task, where each stimulus to be remembered is followed by one or more distractors to be ignored, performance is typically inferior when these distractors are unique (Lewandowsky & Oberauer, 2015). The explanation for this is simple: Novel distractors are—unintentionally—encoded into WM with greater strength, and thus interfere with the representations of information an individual wishes to remember.

Models sharing characteristics with SOB have recently been applied to visual WM data as well, with considerable success (e.g., Oberauer & Lin, 2017; Peteranderl & Oberauer, 2017), indicating that a single framework might be used for modelling both verbal and visual WM. For instance, the interference model (IM) proposed by Oberauer and Lin (2017) involves bindings between contents (in this case, colors) and contexts (in this case, spatial locations) which are represented as a two-dimensional weight matrix, as is the case for the storage of word-position bindings in SOB. The IM provided a superior account of a number of phenomena observed in continuous reproduction tasks (e.g., Zhang & Luck, 2008) when compared to competing explanatory models (e.g., Bays & Husain, 2008; van den Berg, Shin, Chou, George, & Ma, 2012; Zhang & Luck, 2008). However, one feature of SOB that was absent in the IM was novelty-dependent encoding: The task that (Oberauer & Lin, 2017) were modeling did not involve any manipulations of novelty, or variations in encoding time, making the inclusion of encoding rate in the model unnecessary. This leaves open the question of whether novelty, and specifically the novelty of distracting information, has similar effects on performance in visual WM as it does in verbal WM.

Assuming that verbal and visual WM operate according to similar principles, distracting information should impinge more heavily on the existing contents of visual WM when it is novel than when it is not. However, some findings in the visual WM literature can be interpreted as contradicting this principle. For instance, a series of studies undertaken by Allen and colleagues (Allen et al., 2014; Hu, Hitch, Baddeley, Zhang, & Allen, 2014; Hu, Allen, Baddeley, & Hitch, 2016; Ueno, Allen, Baddeley, Hitch, & Saito, 2011a; Ueno, Mate, Allen, Hitch, & Baddeley, 2011b), in which visual “suffixes”—task-irrelevant stimuli presented following the offset of task-relevant stimuli—led to a decrease in the accuracy of recognition and recall, showed that interference was particularly strong when suffixes were plausible. Plausible suffixes took features (e.g., color, shape) from the same pool as the items that needed to be remembered, which meant that these features were viewed more frequently than those possessed by implausible suffixes. As such, the novelty value of the implausible suffixes should have been greater, yet these suffixes produced less interference.

Another reason to think that novelty may produce different effects in visual WM than in verbal WM relates to the features of visual environments themselves, which distinguish them from verbal or auditory environments. Objects tend to persist in the visual environment in a way that sounds often do not. For instance, whereas hearing the drumming of a snipe suddenly cease as the bird changes trajectory in the sky would not be a particularly surprising experience, the same could not be said were the snipe suddenly to blink out of visual existence mid-flight. Nonetheless, the visual signals objects produce do change as we, and the objects, move. To retain a coherent perceptual experience, we must update object representations in accordance with the new input. This idea underlies the concept of object files (e.g., Kahneman, Treisman, & Gibbs, 1992), defined as temporary representations of visual objects that can be updated as input changes. The reviewing process by which this takes place is facilitated by similarity—particularly locational similarity—between the current and previous states of an object. When the two states are similar, the object file is likely to be updated with the new value; whereas when they are different, a new object file is required (Kahneman et al., 1992). In this framework, then, a distractor that is similar to the current contents of memory should be more likely to lead to an update of those contents than one that is wholly novel. This thus has the potential to result in greater interference from *less* novel distractors, which would more frequently replace the prior contents of memory.

Further complicating matters, a series of studies by Fiacconi and colleagues (e.g., Cali, Fiacconi, & Milliken, 2015; Fiacconi & Milliken, 2012, 2013; Fiacconi et al., 2020) has shown that distractors which share identities with the items in a memory array, but are bound to different locations, are more disruptive to memory than distractors that either repeat the memory array’s identity–location combinations, or are entirely novel. The procedure in these studies required participants to remember an array, consisting of letters presented in two of four possible locations, while a second array of 1–2 letters was presented during the retention interval. When an item in the second array required a response, memory for the identity–location binding of items from the initial array became worse. The effect was most pronounced in cases where the identity–location binding of the target item in the second array involved a “feature switch” relative to the initial array—for instance, when an X was presented above fixation in the first array, and to the right of fixation in the second.

In sum, analogies between visual and verbal WM, linked to successful models of these systems, suggest that the novelty of distracting information should be positively related to the interference it produces. On the other hand, one interpretation of research on the effects of visual suffixes, and the greater facility with which similar stimuli lead to updates of object representations, suggest the opposite conclusion. Further, Fiacconi and colleagues’ studies suggest that novelty and interference may have a more complex relationship than either of these possibilities, with novel bindings of non-novel features causing the most memory disruption. It was this uncertainty about the impact of novelty on interference in visual WM that I aimed to resolve with the experiments reported here. Next, I describe what these experiments entailed, and how they would shed light on this issue.

## The present study

There were two related questions I aimed to address in this study. First, does the novelty of distracting information have a detrimental effect on the existing contents of memory (relative to non-novel distractors)? And, second, how do different types of novelty—novelty of features vs. novelty of bindings—moderate any effect?

Based on the literature described above, I can derive two qualitatively different predictions concerning the answers to these questions. First, if novelty in visual WM operates in a similar way as is suggested in SOB and related models of verbal WM (e.g., Farrell & Lewandowsky, 2002; Oberauer et al.,, 2012; Lewandowsky & Oberauer, 2015; Oberauer & Lewandowsky, 2014), then memory performance ought to be worse when information needing to be remembered is subject to distraction from novel stimuli relative to non-novel distractors. Additionally, because novelty in these models is based on similarity between the binding of a distractor to the bindings already held in WM, then distractors containing entirely novel features, or simply novel bindings of existing features, should both be more disruptive than a distractor that involves repeating an existing binding.

Second, if similarity between distractors and the existing contents of memory increases the probability that those contents are updated or replaced with the distracting information, then memory performance ought to be better when distraction comes from novel stimuli than when it comes from stimuli that are more similar. In particular, novel features should be less likely to force an update to the contents of memory than non-novel features. Of course, a non-novel feature presented in a non-novel location (i.e., a distractor that is identical to something that is already in memory) ought not to impede memory performance even if an update occurs, because the old and updated values would be the same. However, non-novel features presented in new locations (i.e., new bindings of old features) should be more likely to produce an update than novel features presented anywhere (i.e., new bindings of new features).

Note that the latter of these predictions essentially reflects the pattern of data reported in the studies by Fiacconi and colleagues (e.g., Cali, Fiacconi, & Milliken, 2015; Fiacconi & Milliken, 2012, 2013; Fiacconi et al., 2020), potentially indicating that distractor novelty in verbal and visual WM has different implications. There are reasons why the story might not be so simple, though. First, in their studies, the array presented during the retention interval required a response using the same stimulus-response key mappings as were used for the subsequent responses to the memory array. In addition, interference was either non-existent, or substantially reduced, when no response to the distractor array was required. This could indicate that the interference occurred at the level of bindings between visual WM representations and responses, rather than within bindings between visual features (e.g., location and identity) themselves. On the other hand, it may simply reflect an increased probability of encoding distractor stimuli into visual WM—leading to interference from these stimuli—when they require a response than when they do not. Second, the brief (157 ms) presentation of distractor arrays likely necessitated their encoding into WM so that participants could respond to them. It is not clear whether stimuli that remain perceptually available would produce a similar pattern of interference effects: their ongoing perceptual availability may remove the necessity to encode them into WM to allow for a suitable response. Third, the stimulus set used in these studies was limited to a minimum of two and a maximum of four stimuli across experiments. This raises questions about how much the distractor stimuli across conditions actually differed in novelty, given that even stimuli which had not appeared in the memory array on a particular trial would likely have been seen quite recently anyway.^1^

In light of these issues, the method I chose to investigate the effect of novelty on distraction in visual WM involved a combination of aspects taken from tasks used in previous studies, along with some additional modifications. Both of the experiments reported here used variations of a single procedure, involving a combination of change detection and recall tasks. First, participants viewed a three-item visual array. Second, they had to respond to a single-item, location-specific change-detection probe, and indicate whether or not it matched the array item that had been presented at that location. Third, participants’ memory of a stochastically independent item from the initial array was tested using a continuous reproduction task. My primary interest was in performance on this final recall test, and how this was affected by different types of change detection probes presented in the second stage. To assess the effects of distractor novelty, I compared recall performance following the presentation of three different types of change detection probes: positive probes, which match the tested item and are novel in neither features nor bindings; negative probes, which do not match the tested item and are novel in both features and bindings; and intrusion probes, which do not match the tested item and are novel in bindings, but non-novel in features.

The use of different response methods for the change detection (binary key-press) and recall (mouse response on a continuous scale) phases of each trial meant that the possibility of confusion between stimulus-response bindings during these phases should have been limited. Additionally, as the change detection probe remained on-screen until the participant produced a response, this removed the participant’s necessity to encode the stimulus into visual WM to complete the task. Further, since stimulus feature values were drawn from a quasi-continuous space with 360 possible values, there was a very low probability that identical values would be repeated across consecutive trials, allowing for a greater novelty difference between trials with change detection probes that repeated a feature from the initial memory array, and those with features not contained in that array (relative to the use of a small set of frequently repeated stimuli).

To preview the main findings, in Experiment 2, results showed that novel change detection probes led to worse recall, even when a different location was probed than was tested in the recall phase. However, the data were ambiguous concerning the level of novelty at which this effect occurred. Experiment 2 clarified this issue, showing that binding novelty was sufficient to produce the effect, and that it did not depend on memory access during the initial phase. These results suggest that novelty-gated encoding is a feature of visual WM as well as verbal WM.

## Experiment 1

In Experiment 2, participants memorized briefly presented arrays of three colored circles. In one condition (change detection and recall, hence CD+R), their memory was subsequently tested using both change detection and recall probes, with the latter requiring them to reproduce the value of the probed item using a color wheel. In another condition (recall-only, hence R), participants only had to recall the value of the probed item (i.e., there was no preceding change detection test).

On CD+R trials, the items probed in the two phases were stochastically independent. This prevented the change detection probe from providing evidence regarding the item likely to be tested in the recall phase; that is, it prevented the probe from acting as an informative retro-cue. There is already substantial evidence indicating that informative retro-cues enhance performance (for review, see Souza & Oberauer, 2016), but here I was primarily interested in the effect of different change detection probe types on recall, rather than in the effects of anything resembling a retro-cue.

I assessed effects of novelty on distractor interference by comparing recall performance following positive, negative, and intrusion change detection probes. If novelty has a deleterious effect, recall should be better following positive probes than following negative or intrusion probes. On the other hand, if similarity has a deleterious effect, the opposite should be true. Additionally, negative and intrusion change detection probes are novel in different ways: For negative probes, both feature and binding are novel, whereas for intrusion probes, the binding is novel but the feature is not. Thus, comparing trials with all three change detection probe types should allow a determination what sort of novelty matters, if indeed novelty has an effect.

### Method

#### Participants

Sixteen participants (mean age 25 years, range 19–33; 14 female, two male), recruited from the University of Zurich (UZH) community via a mailing list, completed the experiment, and were compensated with their choice of CHF15 per session or (if applicable) credit toward their psychology classes. Fifteen participants completed three sessions each, and one participant (who initially misunderstood the instructions) completed four, with data from their first session discarded. I chose the sample size on the basis of prior experience with similar experiments, showing that a sample of this size is typically sufficient to detect meaningful within-subjects effects when each participant completes multiple sessions.^2^

#### Stimuli and apparatus

The experimental task was programmed and executed in PsychoPy (Peirce, 2007; Peirce, Gray, Simpson, MacAskill, Hȯchenberger, & Sogo, 2019) running under Windows 10. The program was displayed full-screen on 27-inch Benq Zowie XL LCD monitors with screen resolution 1920 × 1080 and a refresh rate of 144 Hz. Participants responded to change detection probes with the “<” and “-” keys on a standard Swiss German keyboard (i.e., the locations of the “z” and “/” keys on a QWERTY keyboard), and responded to recall probes using the mouse.

Memory arrays consisted of three colored circles with radius 75 pixels (approximately 2^∘^ of visual angle with 60-cm viewing distance), presented in an equilateral triangular configuration with 346 pixels (approximately 10^∘^ of visual angle) between the center of any two stimuli. On each trial, the colors themselves were taken from 360 equidistant azimuth values of an isoluminant DKL color wheel with elevation 0 and contrast 1 (Derrington, Krauskopf, & Lennie, 1984). To ensure two items never had identical colors, a single value was drawn from a uniform distribution bounded at 1 and 360, and one of the three items given this azimuth value; the other two items were offset by unique values randomly sampled (without replacement) from a set of 17 values between 20 and 340 at intervals of 20. Change detection probes were identical in size, and took values from the same color space: Positive probes were identical to the item that had been presented in the same location; intrusion probes were identical to an item that had been presented in another location; and negative probes took one of the 15 remaining offset values from the set described earlier. Recall probes were white-outlined circles that corresponded to the size and location of the tested memory item, and the color wheel used to record recall responses corresponded to the space from which the item colors were sampled, and was randomly rotated on each trial. Stimulus masks were random color grids with the same shape, size, and location as the memory items.

#### Procedure

**Fig. 1:**
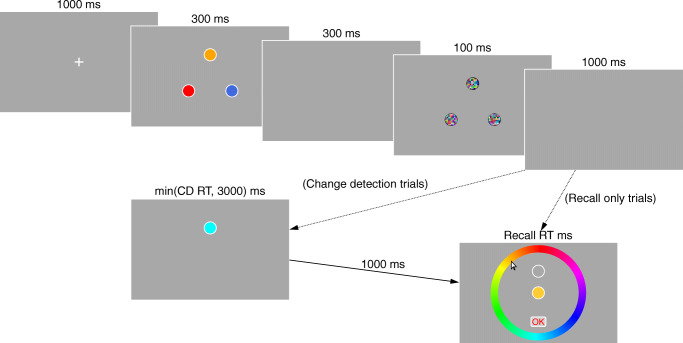
Example of progression of events within trials in Experiment 2. On trials with both change detection and recall tests, the post-mask interval was followed by the presentation of a change detection probe; participants had to make a manual response indicating whether the probe matched the item previously presented in that position. This was followed by a recall test of an independently sampled item (here, the same item is tested in change detection and recall tests). In recall-only trials, the post-mask interval was immediately followed by the recall test. Note that the figure uses colors from an HSV color space. whereas colors in the experiment itself came from a DKL space

Figure 1 shows an example of the progression of events within a trial. Participants completed two types of blocks: CD+R blocks and R blocks. Both block types began identically, with a central fixation cross presented for 1000 ms. This was followed by the presentation of a three-item array for 300 ms, a 300-ms blank period, a 100-ms mask, and a 1000-ms retention interval. Following the retention interval, the block types diverged. In CD+R trials, a single change detection probe was presented in one of the three positions previously occupied by an item from the memory array. There were three probe types in this phase. Positive probes were identical to the item presented in the same location in the memory array, and required a “match” response. Intrusion probes were the same color as an item presented in a different location in the memory array, and required a “mismatch” response. Finally, negative probes differed in color from all three items that had been presented in the memory array, and also required a “mismatch” response. Participants had up to 3000 ms to make a manual response (response assignment counter-balanced across participants) indicating whether or not the item was the same as or different to the memory item that had been presented in the same location. Once they had made their response, or 3000 ms had elapsed, there was a further 1000-ms blank interval. This was followed by the presentation of a recall probe, in the form of a white circular outline in one of the three memory locations, along with a color wheel. Participants had to click on the color wheel to indicate what color they thought the memory item in the cued position had been. With each click, a centrally displayed circle changed color to show them their selection. Once they were satisfied, they clicked an “OK” button to end the trial. In R trials, there was no change detection test, and the recall test occurred immediately after the 1000-ms retention interval. After the trial ended, there was a 1200-ms inter-trial interval. Participants did not receive any feedback regarding their change detection or recall responses.

Each participant completed three experimental sessions of approximately 1-h duration per session, containing two practice blocks and nine experimental blocks. To begin each session, they completed a practice block of 10 CD+R trials. This was followed by four experimental blocks of CD+R trials, a second practice block of ten R trials, two experimental blocks of R trials, and a further three experimental blocks of CD+R trials. Each experimental block contained 36 trials, so each session contained 252 CD+R trials and 72 R trials. In total, each participant completed 756 CD+R trials and 216 R trials. In the CD+R blocks, $\frac {1}{4}$ of trials included positive probes in the change detection test, $\frac {1}{4}$ of trials included negative probes, and $\frac {1}{2}$ included intrusion probes.^3^

#### Data analysis

I analyzed raw data using the *BayesFactor* R package (Morey & Rouder, 2015). In each case, I aggregated participants’ data to the means for the relevant conditions before conducting these analyses, using the *aggregate* function.^4^

To determine whether change detection probe novelty affected interference, I separately compared recall performance in same- and different-item CD+R trials following positive, negative, and intrusion change detection probes, using two one-way ANOVA, followed by *t* tests where necessary. Though it seemed likely to me that any interference effects would be most obvious on same-item trials (where the interfering probe is presented in the same location as the subsequently tested item), a comparison between probe types in this condition is subject to problems with target re-presentation: The subsequently tested item is re-presented on same-item positive probe trials, but not on negative or intrusion probe trials. For this reason, one might find a recall advantage following positive change detection probes simply because the participant has seen the target item twice, rather than because non-novel probes produce less interference. Because of this, I also compared performance on different-item trials following the three probe types, after removing data from intrusion probe trials where the intrusion came from the item that was subsequently tested at recall (i.e., those that involved the re-presentation of the target feature, albeit at a different location). A difference between the change detection probe types in both same-item and different-item trials would thus provide strong evidence concerning the effects of novelty on interference, as well as indicating that such effects are not limited to memory for the item in the location where the interfering stimulus is presented.^5^

In addition to the raw data analyses, I also fit the recall distributions with mixture models (e.g., Bays, Catalao, & Husain, 2009; Zhang & Luck, 2008), to provide information about the source of any effects of retrieval practice and interference on recall. With one exception, these analyses were not particularly illuminating, so I describe the relevant method and results in the Supplemental Materials. Data from the R trials are relevant to a separate and ongoing project concerning testing effects in visual WM, so I do not report any analyses for them. For the same reason, I do not directly compare responses in same-item and different-item CD+R trials.

I report Bayes factors (*BF* s) for all statistical tests such that values > 1 imply evidence in favor of a difference (or specified model), and values < 1 imply evidence against a difference (or specified model); values close to 1 represent ambiguous evidence. Though Bayes factors provide a continuous measure of evidential strength (i.e., there is no customary “significance” criterion), various rules-of-thumb for their interpretation have been suggested. Here, I follow the guidelines provided by Kass and Raftery (1995), in treating *BF* values from 0.33 to 1 and 1 to 3 as providing only weak evidence against and in favor of a difference, respectively.

### Results

One participant’s recall responses approximated the error levels associated with random responding (average absolute error approximately 85^∘^, where completely random responding would result in mean error of 90^∘^), so I removed their data from all analyses.

#### Does distractor novelty affect recall?

##### Omnibus analysis

**Fig. 2:**
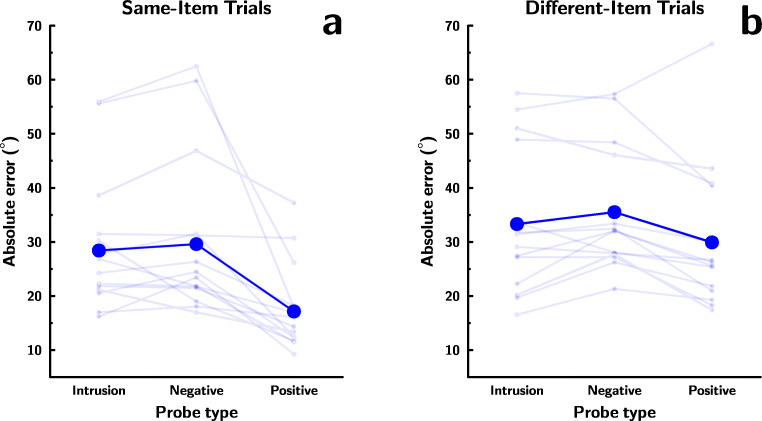
**a** Recall performance in same-item CD+R trials in Experiment 2, following intrusion, negative, and positive change detection probes. **b** Recall performance in different-item CD+R trials following intrusion, negative and positive change detection probes (with data from trials with intrusions from the subsequently tested recall target removed). In both panels, *larger*, opaque*points* (*and associated lines*) reflect grand means; *smaller*, *semi-transparent points* (*and associated lines*) are means of individual participants

Figure 2 shows recall error for CD+R trials, with panel (a) showing data from same-item trials, and panel (b) data from different-item trials. Recall was superior in same-item than different item trials, superior following positive than negative or intrusion change detection probes, and the latter difference was more pronounced in same-item than in different-item trials. As a result, the best model in the omnibus ANOVA contained both main effects and their interaction (*BF* = 3.14 relative to the second-best model, containing both main effects but omitting the interaction).

##### Same-item trials

Panel (a) of Fig. 2 shows recall error for CD+R trials where the same item was tested in the change detection and recall phases, separately for trials with the three different change detection probe types. Change detection probe type affected performance (*BF* = 534.70 for model with vs. without main effect of probe type): Recall error was lower following a positive probe than following a negative probe (*BF* = 31.88) or an intrusion probe (*BF* = 36.91). This is consistent with the idea that novel distractors interfere more with the contents of visual WM; however, as noted earlier, this could also result from better performance following re-presentation of the tested feature in positive probe trials. Performance following negative and intrusion probes was roughly equivalent (*BF* = 0.37).

##### Different-item trials

Panel (b) of Fig. 2 shows recall error for CD+R trials where a different item was tested in the change detection and recall phases, separately for trials with the three different change detection probe types. As was the case for same-item trials, change detection probe type affected recall performance (*BF* = 10.14 for model with vs. without main effect of probe type): Recall error was lower following positive probes than following negative probes (*BF* = 18.00), but the evidence was ambiguous regarding differences between recall following intrusion probes and positive probes (*BF* = 1.03) or negative probes (*BF* = 1.05). This is consistent with the idea that novel distractors interfere more with the contents of visual WM, but leaves ambiguous the question of whether presenting an entirely new feature is necessary to cause greater interference.

### Discussion

The results of Experiment 2 lead to one clear conclusion, with the answer to a second question remaining unclear. Specifically, both same-item and different-item trials showed worse performance following novel than non-novel change detection probes. This is consistent with the idea that stimuli are encoded into visual WM with a strength dependent on their novelty (i.e., novelty-gated encoding; i.e., novelty-gated encoding; e.g., Farrell & Lewandowsky, 2002; Oberauer & Lewandowsky, 2013, 2014). However, less clear was whether the level or type of novelty moderates this effect. For same-item trials, there was evidence against a difference in recall performance following negative and intrusion change detection probes; whereas for different-item trials, the evidence was ambiguous. Overall, however, the general pattern of the data across both same- and different-item trials was consistent.

It is also worth considering the possibility that the interference evident in Experiment 2 resulted from participants’ need to access WM when completing the change detection test. Evidence from behavioral research with humans, and behavioral and pharmacological research with non-human animals, has shown that retrieving previously consolidated memories can lead them to revert to a labile state, increasing effects of interference or extinguishing the initial memories altogether (e.g., Hupbach, Gomez, Hardt, & Nadel, 2007; Nader, Schafe, & Doux, 2000). Though these findings pertain to long-term memory, it is not clear what effect the use of WM has on its stability. I addressed this question, and attempted to shed further light on what sort of novelty is required for stimuli to interfere with the contents of visual WM, in Experiment 2.

## Experiment 2

Experiment 2 was mostly identical to its predecessor: A three-item array was briefly presented, recall for one of the items was tested on every trial, and in one block type the recall test was preceded by a change detection test of a stochastically independent item. The major difference was that those blocks without change detection included an alternative task prior to recall, with identical stimuli and manual responses across the two block types. Specifically, in this novel task participants had to decide whether probe stimuli presented in the first phase—oriented arrows—were pointing to the left or to the right: a “direction judgement” (DJ) task. This modification was designed to allow for a comparison of the effect of distracting stimuli on performance between trials with and without memory access (i.e., pre-recall retrieval). I also used a slightly greater number of participants, in the hope that collecting a greater amount of data would resolve any ambiguities in the results of the previous experiment.

### Method

Unless otherwise noted, methodological details in Experiment 2 were identical to those in Experiment 2.

#### Participants

Twenty-four participants (mean age 21.5 years, range 18–32; 20 female, four male) each completed three experimental sessions. I used a larger sample size in this experiment in the hope that the analyses would produce less ambiguous results regarding differences between the two types of novel probes.

#### Stimuli and apparatus

Participants responded to change detection and binary direction judgement probes with the “<” and “x” keys on a standard Swiss German keyboard (corresponding to the locations of the “z” and “c” keys on a QWERTY keyboard). This change was designed to allow them to make keyboard responses with separate fingers of the left hand and mouse responses with the right, without having to shift either hand between the keyboard and the mouse during a trial.

**Fig. 3:**
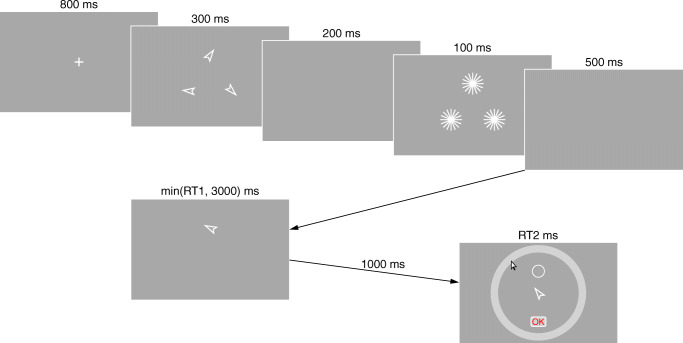
Example of progression of events within trials in Experiment 2. After stimulus presentation and masking, participants completed either a change detection or direction judgement test, with identical stimuli used for both tasks. The first task was followed by a recall test of an independently sampled item (here, the same item is tested in change detection/direction judgement and recall tests)

Memory arrays, as well as change detection and direction judgement probes, consisted of white-outlined arrows (see Fig. 3) with a maximum height of 100 pixels (approximately 3^∘^ of visual angle) and a maximum width of 60 pixels (approximately 2^∘^). Their orientations were selected in the same way that azimuth values were selected for the color stimuli in Experiment 2. Memory arrays were backward-masked by 20-point “stars”, with each point extending from the center of the stimulus location for 80 pixels (approximately 2^∘^ of visual angle). A solid light grey orientation wheel was used to record recall responses; participants clicked on the location corresponding to their memory of the direction in which the arrow being tested had pointed.

#### Procedure

Figure 3 shows example trial progression. Because every trial in Experiment 2 involved two tasks, I reduced the duration of some events relative to Experiment 2 to allow participants to complete a similar number of trials without having to extend session time. Specifically, I reduced the presentation time for the fixation cross from 1000 to 800 ms, the pre-mask interval from 300 to 200 ms, the retention interval preceding the first task from 1000 to 500 ms, and the inter-trial interval from 1200 to 1000 ms. In each session, participants completed 12 blocks of 27 experimental trials each, for a total of 324 trials per session (972 trials per participant). In addition, participants completed a short practice block prior to beginning the experimental trials, and prior to switching from change-detection to direction-judgement blocks. Each session began with four CD+R blocks, followed by four direction-judgement blocks (hence, DJ+R), and concluded with another four CD+R blocks. To balance a desire for roughly equal response frequencies in the CD/DJ tasks with a desire to have each change detection probe type roughly equally presented, 31*%* of trials used negative probes in the initial test, 31*%* used intrusion probes, and 38*%* used positive probes. Identical proportions of these stimuli were also used in DJ+R trials; though the task did not involve comparison of these probes to the items in memory, I use the same names to describe the probes because the conditions are otherwise the same (e.g., a “positive probe” in the direction judgement task was identical to the stimulus presented at the same location in the memory array, but participants simply judged whether it was pointing left or right). In all other respects, the procedure was identical to that of Experiment 2.

#### Data analysis

Data analysis proceeded similarly to that in Experiment 2. To assess the effects of distractor novelty on interference, I conducted separate two-way ANOVA for same-item and different item trials, using the factors probe type (intrusion, negative, or positive) and block type (CD+R or DJ+R). If there were an effect of novelty, this should show up as a main effect of probe type in each ANOVA, which could be followed by paired comparisons to confirm the source of the effect. Further, if this effect were moderated by whether or not the interfering stimulus had to be compared to the contents of memory, this should lead to an interaction between change detection probe type and block type. To avoid extraneous effects that might result from presenting the target twice, I excluded different-item intrusion trials where the intrusion came from the item subsequently tested.^6^

As for Experiment 2, I supplemented these analyses with mixture modeling. However, there were too few trials per participant in some of the direction judgement conditions to allow for valid parameter estimates. Consequently, I collapsed data across CD+R and DJ+R trials. I report the relevant analyses in the Supplemental Materials.

### Results

Due to a programming error, keyboard responses for three participants’ first sessions were not properly recorded, so the data were not usable. I also excluded data from two participants from all analyses due to unusually high average recall error (78 and 80^∘^, where completely random responding results in mean error of 90^∘^, and other participants’ averages were between 14 and 53^∘^).

#### Does distractor novelty affect recall?

##### Same-item trials

**Fig. 4:**
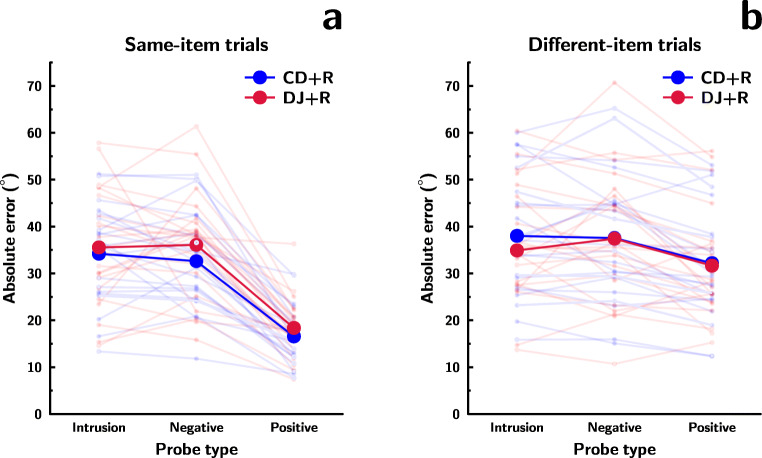
**a** Recall performance in same-item CD+R and DJ+R trials in Experiment 2, following intrusion, negative, and positive first task probes. **b** Recall performance in different-item CD+R and DJ+R trials following intrusion, negative and positive first task probes (with data from trials with intrusions from the subsequently tested recall target removed). In both panels, *larger*, *opaque points* (*and associated lines*) reflect grand means; *smaller*, *semi-transparent points* (*and associated lines*) are means of individual participants.

Panel (a) of Fig. 4 shows recall error for CD+R and DJ+R trials where the same item was tested in both phases, separately for trials with the three different types of change detection probes. Recall performance was affected by probe type, and the best model included only this main effect (*BF* = 2.85 × 10^21^, relative to the intercept-only model). Evidence against the model with an added main effect of block type was minuscule (*BF* = 0.99, relative to the best model). However, both were superior to the model containing an interaction between the two factors in addition to the main effects (both *BF* > 6.09). Consequently, I used data from both block types in paired comparisons between trials involving the different change detection probe types. Recall error was lower following positive probes (mean = 17.18^∘^) than following either negative probes (mean = 33.78^∘^; *BF* = 4653130) or intrusion probes (mean = 34.64^∘^; *BF* = 3207174). This is consistent with the idea that novel distractors interfere more with the contents of visual WM. However, as noted earlier, this difference could also result from better recall following positive change detection probes due to re-presentation of the subsequently tested feature. Performance following negative and intrusion probes was roughly equivalent (*BF* = 0.25).

##### Different-item trials

Panel (b) of Fig. 4 shows recall error for CD+R and DJ+R different-item trials, separately for trials with the three types of first task probes. Recall was affected by change detection probe type, with the best model having a main effect of probe type only (*BF* = 962.27 relative to the intercept-only model). This model was slightly superior to the model that also included a main effect of block type (*BF* = 2.35), and also superior to the model that included a main effect of probe type and a probe type × block type interaction (*BF* = 3.97) and the model that included both main effects and the interaction (*BF* = 10.84). Consequently, I combined data from both block types and used *t* tests to compare performance following the different change detection probe types. Recall error was lower following positive probes (mean = 32.02^∘^) than following either negative (mean = 37.52^∘^; *BF* = 1193.53) or intrusion probes (mean = 37.00^∘^; *BF* = 471.08), but performance following negative and intrusion probes was roughly equivalent (*BF* = 0.25). This is consistent with the idea that novel distractors interfere more with the contents of visual WM, and that binding novelty alone is sufficient to produce this effect.

### Discussion

The results of Experiment 2 provided some clarity after the slightly ambiguous findings from Experiment 2, producing similar data patterns to that experiment but with stronger statistical evidence, likely as a result of the increased sample size. For both same-item and different-item trials, recall performance was better following a non-novel distractor (i.e., a positive change detection probe) than following either type of novel distractor (i.e., negative and intrusion change detection probes). Further, performance following the two types of novel distractors was roughly equivalent, indicating that novel distractors have the same interfering effects irrespective of whether they involve the presentation of an entirely new feature, or simply the re-arrangement of features that are bound differently in the existing contents of memory.

Though there was only weak evidence against a difference between performance in the change detection and direction judgement blocks, the general patterns across both were similar. Thus, it does not appear that comparison to the existing contents of memory is required to produce the distracting effects of novel stimuli; rather, they are a result of the stimuli themselves.

## General discussion

Through these experiments, I aimed to determine whether distractors’ novelty affects the extent to which they interfere with the contents of visual WM. Based on the results, the answer to this question appears to be “yes”. Both experiments reported here showed clear effects of distractor novelty on subsequent recall: Recall error was higher following negative or intrusion change detection probes than following positive probes. This was true even when a different item was probed in the first phase than was subsequently tested in the recall phase, which rules out the possibility that the effect was an artefact of positive probes providing an extra study opportunity for the tested stimulus. It was also true whether the probes were part of a task that required memory access (i.e., change detection), or one that was purely perceptual (i.e., direction judgement). Next, I discuss what these results imply for the two questions I set out to address in this study: “How does novelty affect distractor interference?”, and “How do different types of novelty moderate this effect?”. I conclude by considering some additional implications of these results.

### How does novelty affect distractor interference?

In the Introduction, I highlighted two plausible ways that distractor novelty might affect interference with the existing contents of visual WM. First, consistent with the implementation of novelty-gated encoding in a prominent computational model of working memory (SOB, and its off-shoots; e.g., Farrell & Lewandowsky, 2002; Oberauer & Lewandowsky, 2013, 2014) the stronger encoding of more novel distractors should produce greater interference with the contents of WM. Second, consistent with findings from studies using visual suffixes (e.g., Allen et al., 2014; Hu et al., 2014, 2016; Ueno et al., 2011a, 2011b), examining the circumstances under which object files are reviewed (Kahneman et al., 1992), and exploring interactions between consecutive arrays (e.g., Cali et al.,, 2015; Fiacconi & Milliken, 2012; (e.g., Cali, Fiacconi, & Milliken, 2015; Fiacconi & Milliken, 2012, 2013; Fiacconi et al., 2020), stimuli that are more similar to the current contents of visual WM may be more likely to cause an update of existing bindings, producing an inverse relationship between distractor novelty and interference. The results of the experiments reported here are most consistent with the former account: Novel distractors, presented as probes in a task during the retention interval, led to worse recall of memory stimuli. This lends support to the idea that novelty-gated encoding is a consistent principle across both verbal and visual WM.

How can this conclusion be reconciled with findings that might be interpreted as suggesting the opposite relationship between novelty and interference—or at least, a relationship that is more complicated? First, Allen and colleagues (e.g., Allen et al., 2014; Hu et al., 2014, 2016; Ueno et al., 2011a, 2011b) found that plausible visual suffixes led to greater interference than implausible suffixes; since plausible suffixes involved features that were more frequently repeated (and thus likely more expected) than implausible suffixes, this could be interpreted as indicating greater interference by *less* novel stimuli. However, there are a number of differences between these studies and the experiments reported here that may be relevant. For one, whereas the distractor stimuli in the present experiments were always task-relevant, and thus could not be filtered out, this was not true of the suffixes in the earlier experiments. For instance, Ueno et al., (2011b) suggested that an attentional filter, used to discriminate valid and invalid stimuli on the basis of the features they possessed, could account for the greater interference produced by plausible suffixes in their experiments. Thus, even though implausible suffixes may have been more novel, they could typically be filtered out prior to any impact on the contents of memory, whereas plausible suffixes could not. Additionally, there was no requirement for participants in the suffix studies to respond to those stimuli, whereas in the present experiments the distractors always required a response. Responding to distractor stimuli seems to moderate the extent to which they interfere with memory, with reduced interference when no response is required (e.g., Fiacconi and Milliken, 2012, 2013; Fiacconi et al., 2020).

Second, studies by Fiacconi and colleagues (e.g., Cali, Fiacconi, & Milliken, 2015; Fiacconi & Milliken, 2012, 2013; Fiacconi et al., 2020) consistently found greater interference from intervening arrays that shared features with items to be remembered. For example, Cali et al., (2015) found that distractors produced more pronounced interference when they involved a feature switch (i.e., an “old” identity presented in a different location, roughly equivalent to the intrusion change detection probes in my experiments) than when they involved the presentation of a stimulus that had not appeared in the first array (roughly equivalent to the negative change detection probes in my experiments); yet here this pattern was not evident. One explanation for this apparent discrepancy may relate to the small set of easily identifiable stimuli used in those experiments, and the use of only two memory stimuli on each trial. If participants retain representations of the identities and locations of stimuli presented in the memory array, even when the bindings between these features are lost—as suggested by Cali et al., (2015)—this may allow them to respond to a memory test with an informed guess about the nature of the relevant binding (see, e.g., Rhodes, Cowan, Hardman, & Logie, 2018 for evidence of informed guessing in tests of visual WM). Further, if the distractor stimulus is sometimes mistaken for a memory stimulus, this would reduce the success of such a guessing process. To provide a concrete example, consider a hypothetical trial from one of Cali et al.,’s experiments where the initial array consists of the letter “A” presented above fixation (position 1), and the letter “B” to the right (position 2). If the hypothetical trial is from the feature switch condition, the distractor array could then consist of the letter “B” presented above fixation (position 1). At the memory test, the participant is then probed with the letter “A” and asked to recall the location in which it was presented. If they have retained the initially formed identity–location bindings, they can correctly respond that this stimulus was presented in position 1. On the other hand, if they have lost the bindings but retained memory of the features, they will know that the letters A and B were presented, and that one of them was presented in location 1 and the other in location 2, but not which was presented where. With no further information available, they can, at best, respond with either of the two possible locations equiprobably. If they mistake the distractor for one of the memory stimuli, however, and believe that the “B” was presented in position 1, they will mistakenly infer that the “A” was presented in the remaining position 2, leading to an informed guess that produces worse-than-chance performance. By contrast, in cases where the distractor is not a plausible memory item—because it mismatches the identities or locations retained in feature memory (or both)—the guessing will revert to equiprobability between both possible response options. Overall, this process would lead to worse performance in a feature switch trial than in a trial with a distractor with features that were absent from the memory array. By contrast, in the experiments reported here, with three memory stimuli per trial that lacked discrete identities, both the process of rejecting distractor stimuli that mismatch the features of the original memory array items, and the inferences necessary for informed guessing, would be more difficult, possibly to the extent that participants would not attempt such a strategy.

Following on from this point, in the present experiments, if participants sometimes failed to retain the original bindings, and then confused change detection/direction judgement probes for memory stimuli, this should have had a different effect in same-item trials (where the change detection probe was presented in a location subsequently tested) than in different-item trials (where the change detection probe was presented in a location not subsequently tested). Specifically, we would expect them to mistakenly recall the probe value with a greater frequency in same-item trials than in different-item trials. Results from the mixture modeling in Experiment 2 (see Supplementary Materials) show exactly this pattern: On same-item trials, participants reported the change detection probe values on a substantial minority of occasions and randomly guessed rarely, whereas on different-item trials, random guesses were more frequent and probe reports relatively rare. Thus, the proportion of responses reflecting something other than memory of the target was similar across conditions, but what participants did when they erred differed, because the change detection probes provided a plausible response on same-item trials, but not on different-item trials.

### Effects of binding vs. feature novelty

In both Experiments 2 and 2, recall error in same-item trials following negative and intrusion change detection probes was roughly equivalent (i.e., the Bayes factor in the comparisons favored the null hypothesis). In different-item trials, evidence from Experiment 2 was inconclusive, whereas there was evidence against any difference in Experiment 2. In combination, these results suggest that both types of novel probes interfered with the existing contents of memory to a similar extent. As described in the Introduction, negative change detection probes consist of an item that was not in the memory array, and thus comprise both a novel feature (color in Experiment 2, orientation in Experiment 2), and a novel binding of this feature to a location; whereas intrusion change detection probes consist of an item from the memory array presented at a different location, and thus comprise a non-novel feature with a novel binding. These results thus imply that novelty of features has limited or no additional effect on interference beyond that produced by a novel binding. This is consistent with the form of novelty-gated encoding used in SOB and related models of WM (e.g., Farrell & Lewandowsky, 2002; Oberauer et al.,, 2012; Lewandowsky & Oberauer, 2015; Oberauer & Lewandowsky, 2014), in which novelty is calculated by the comparison of a perceptual stimulus with the *bindings* currently held in memory. In this framework, when the context (e.g., location) in which a perceptual stimulus is presented is distinct from the context to which a stimulus with similar features is bound in WM, the featural similarity between the stimuli becomes irrelevant in this calculation. Along these lines, one can infer that the locations in which stimuli were presented in both of the present experiments were sufficiently distinct from one another that intrusion and negative change detection probes were equivalently novel, and were therefore encoded with greater strength.

In the future, this idea could be directly tested in an experiment where memory stimuli, and probes presented during the retention interval, are unequally spaced (as opposed to their presentation on the vertices of an equilateral triangle in the experiments reported here). Locations that are closer to each other would provide less distinct contexts than those that are more distant. As a result, intrusion change detection probes where the intruding feature comes from a stimulus presented at a nearby location ought to be less novel than probes where the intruding feature comes from a more distant location; this should lead to reduced interference in the former case than in the latter.

### Location and distractor effects

One key result across both experiments was that the change detection probe novelty did not only lead to inferior performance in trials where the same item was tested in both the change detection and recall phases (i.e., same-item trials), but also in trials where items in different locations were tested in each phase (i.e., different-item trials). Superior recall following different-item positive change detection probes, relative to negative or intrusion probes, is important in allowing the inference that probe novelty is responsible for the difference between conditions: Had this difference only been present in same-item trials, it could simply have resulted from re-presentation of the target feature as the positive change detection probe. Nevertheless, evidence of probe interference with memory for items presented at different locations could be interpreted as conflicting with previous findings showing location-specific interference in visual WM. For instance, Makovski and Jiang (2008) showed that proactive interference (i.e., interference by no-longer-relevant information on representations currently subject to test) in a change detection task was only statistically reliable when a probe matched an item presented in the same location on a previous trial; when it matched an item presented in a different location, there was no significant effect. Or, to give another example, the previously discussed studies undertaken by Fiacconi and colleagues (e.g., Cali, Fiacconi, & Milliken, 2015; Fiacconi & Milliken, 2012, 2013; Fiacconi et al., 2020) showed increased interference by distractors when the distractor and tested memory stimulus overlapped. How can this apparent discrepancy be explained?

First, concerning Makovski and Jiang’s (2008) finding of location-specific proactive interference, this could be an outcome of a cue-based search of memory when relevant information is not available in WM (see, e.g., Oberauer, Awh, & Sutterer, 2017, for a similar argument concerning proactive interference and facilitation in visual WM). The relevant experiments involved change detection or 4AFC recognition, with colored circles or novel shapes as memory stimuli, and location-specific probes (i.e., asking participants to decide whether a specified color or shape was presented in a certain location). Proactive interference was evident in more frequent errors when the change detection probe matched the feature value (color/shape) that had been presented in the cued location on the preceding trial, relative to a baseline condition with an entirely novel probe. By contrast, when the probe matched the feature value that had been presented in a different location on the previous trial, no such interference was evident. This pattern would be expected if participants (a) sometimes failed to encode or retain information about the probed item in WM, and (b) searched memory in general (e.g., episodic LTM) in such circumstances using the target location as a cue. Such a search would be more likely to mistakenly turn up the feature presented in that location on the previous trial than to turn up a novel feature, or a feature that had been presented elsewhere on the previous trial. Importantly, this does not imply that the “interfering” item from the previous trial played a role in disrupting WM for the relevant item; just that, when WM was already poor, responses originating in other forms of memory were counterproductive. This is consistent with the fact that, in the experiments reported here, probe intrusions in recall responses were more frequent for same-item than for different-item trials, but that guessing rates showed the opposite pattern: When the relevant information was not available, people erred, but how they responded when they erred differed. This highlights one way that continuous response methods are helpful in understanding patterns of interference: Whereas, in a task with a small and discrete set of responses it is possible for one’s misinformed guesses to lead to worse-than-chance performance, this is less likely when responses are obtained using a color or orientation wheel with hundreds of possible response values and no systematic relationship between target and non-target stimuli. In short, they allow an easier separation of *when* people have a failure of memory, from *what they do* under these circumstances.

Second, concerning Fiacconi and colleagues’ (e.g., Cali, Fiacconi, & Milliken, 2015; Fiacconi & Milliken, 2012, 2013; Fiacconi et al., 2020 consistent finding that interference was greater when distractors overlapped the tested memory stimuli, the conflict between this and the pattern of results in the present experiments may be more specious than genuine. The conditions in their experiments that were most similar to my same-item trials were those in which the distractor stimulus and the memory target occupied the same location. The equivalent to a “positive probe” would thus have been the conditions in which the identity and location of the probe distractor matched those of the memory target (variously labeled “match” or “location–identity” conditions); and the most equivalent to the negative or intrusion probes would have been those in which they did not (variously labeled “feature-switch” or “mismatch” conditions). Comparing these conditions shows almost universally superior performance in the positive probe-equivalent conditions, consistent with the data from my same-item conditions. The conditions in their experiments that were most similar to my different-item trials were those in which the distractor stimulus and the memory target occupied different locations. Here, the patterns from their data are more ambiguous: Comparing match/location-identity conditions (roughly equivalent to positive probe trials) to mismatch/feature-switch conditions (roughly equivalent to negative/intrusion probe trials) shows numerically superior performance for the former in four of the six experiments where the comparison is possible, albeit of a smaller magnitude than that shown in same-item-equivalent trials.^7^ It is not clear that these results are in conflict with mine: My data too show a greater difference between positive and negative/intrusion probes for same-item than for different-item trials.

Fiacconi et al., (2020) also raised the possibility that interference in their experiments reflected a conflict in binding actions (i.e., responses to stimuli in the memory and intervening arrays) to perceptual features. In particular, they referred to work conducted by Stoet and Hommel (1999) showing that associating two stimuli with the same response negatively affects responding to the second such stimulus. In the context of Fiacconi et al.,’s experiments, such a phenomenon would lead to conflict when the stimulus in the intervening display requires the same response as the probed stimulus from the memory array, consistent with their findings of greater RT and memory costs in mismatch trials. As indicated in the Introduction, in the experiments I report here, the response types across the two phases of the task were different—a binary key-press and a continuous mouse response. This may mean that, despite the similarities between the methods used in their experiments and mine, the sources of interference in each case are different, consisting primarily of response conflicts in their experiments, and primarily of perceptual interference in mine. One way to assess this in the future would be to run a modified version of either of the experiments reported here, where the response to the first-phase probe is also given using a mouse-based continuous response scale (e.g., asking participants to select the orientation of the probe arrow, rather than to use it as the basis of a change detection or direction-judgement response). If this produces a greater discrepancy between the interference evident in same-item trials and different-item trials than that found in the present experiments, it would suggest that perceptual interference and response binding conflicts can differently contribute to performance in tests of visual WM.

It is worth noting that the existence of interference effects on different-item trials could also be interpreted as being partially inconsistent with the principles of novelty-gated encoding as incorporated in SOB and related models (e.g., Farrell & Lewandowsky, 2002; Oberauer et al.,, 2012; Lewandowsky & Oberauer, 2015; Oberauer & Lewandowsky, 2014). Whereas novel stimuli should be encoded more strongly than non-novel stimuli in this framework, this encoding involves a binding between the feature of the stimulus (e.g., its color) and the context in which it is presented (e.g., its location). To the extent that distractors are encoded, they will overwrite whatever content is already bound to the context in which they are presented. An implication of this is that interference caused by distractors should only affect features bound to different contexts (e.g., different locations) to the extent that the representations of these contexts overlap with the context in which the distractor is presented. At the very least, this should lead to a greater difference in recall performance following novel vs. non-novel distractors for same-item trials (where the contextual overlap is 100%) than for different-item trials (where it is < 100%); and in cases where the contextual overlap is minimal, interference with memory stimuli presented in different locations should likewise be minimal. Though the data presented here are consistent with the idea that distractor novelty led to greater interference with greater contextual overlap—as evidenced by a greater difference between recall performance following positive vs. negative or intrusion probes in same-item than different-item trials—this may also have resulted from re-presentation of the target feature in same-item positive probe trials, as discussed earlier. As such, an additional mechanism or mechanisms may be necessary to fully account for the effects of distractors on memory for stimuli presented in different locations.

One such mechanism could involve the reviewing of object files. Kahneman et al., (1992) suggested that, in circumstances where a new stimulus cannot be linked to an existing object file in memory, a file is selected randomly for review. In their account, location was the primary feature used to determine which file a new stimulus would be linked to. If this were the case then there is no obvious reason why this process would be different when new stimuli match the color or orientation of the existing object in memory (as in positive change detection probes) compared to when they do not (as in negative or intrusion probes). However, subsequent work suggests that features such as color can also play a role in this selection process (e.g., Moore et al.,, 2010). This raises the possibility that the review of an existing object file at random may be more frequent when the feature-location binding of a stimulus mismatches those held in memory than when it matches (in which case, the matching file is the one reviewed). If so, this would provide a straightforward explanation for greater interference by novel distractors in both same-item and different-item trials: Novel distractors increase the probability that a random object file is reviewed and updated, and therefore decrease the probability that its original feature values are retained.

### Implications of novelty effects on interference for change detection performance

In their studies, Fiacconi and colleagues highlighted the fact that change detection is not a pure measure of visual WM, owing to potential interference from change detection probes (e.g., Fiacconi et al.,, 2020). A similar point has been raised by researchers investigating the effects of retro-cues, who have identified reductions in probe interference as a key contributor to retro-cue benefits (e.g., Makovski, Sussman, & Jiang, 2008; Shepherdson, Oberauer, & Souza, 2018; Souza, Rerko, & Oberauer, 2016). The experiments reported here show that different change detection probe types have different effects on memory. Specifically, they suggest that participants completing change detection tests are less likely to have access to high-quality memory representations on mismatch/change trials than on match/no-change trials, because of the greater interference produced by mismatching probes. Thus, manipulations of variables such as change proportion—usually intended to affect participants’ response bias without changing the quality of memory (e.g., Donkin, Tran, & Nosofsky, 2014; Donkin, Kary, Tahir, & Taylor, 2016; Taylor, Thomson, Sutton, & Donkin, 2017)—may have unintended effects on overall performance. Fortunately, the use of measures from signal detection theory (e.g., *d*^′^, ROCs) or related capacity metrics (e.g., Cowan and Pashler’s *K* s) should be immune to problems in this regard, because they are calculated using hits and false alarms rather than proportion correct. For example, consider a hypothetical experiment with change trial proportions of 0.6 in condition A and 0.4 in condition B. Let participants respond correctly 90% of the time on no-change trials, and 60% of the time on change trials (under the assumption that probes on change trials interfere with memory to a greater extent). In condition A, participants will respond correctly 72% of the time (0.6 × 0.6 + 0.4 × 0.9), whereas in condition B the value will be 78% (0.4 × 0.6 + 0.6 × 0.9). However, the value of *d*^′^ in both conditions will be 1.53 (Φ^− 1^(0.9) −Φ^− 1^(0.4)).^8^

These results also imply that a bias to make a mismatch/change response in situations where little or no information about the probed item is available is optimal. That is, if novel probes interfere more than non-novel probes, a person is more likely to find themselves relying on poor memory for an item following a change than in the absence of one. Thus, it would be strategically wise to make a mismatch/change response under these circumstances. This provides an alternative explanation for the bias to respond “change” that is often present in change detection tasks (e.g., Nosofsky & Donkin, 2016).

The effect of different change detection probe types on memory also raises questions about what constitutes the best measure of a person’s visual WM capacity. Does it reflect the information they retain under conditions that facilitate memory, or under conditions that interfere with it? This brings to mind the distinction drawn in verbal WM between simple and complex span performance (e.g., Turner and Engle, 1989; Unsworth & Engle, 2006, 2007). If there is a difference between how well people respond to novel and non-novel probes, it may be interesting to see whether individual differences in these two contexts are related to performance on more complex cognitive tasks to the same extent.

Finally, the extent to which the interference found here extends to tests of visual WM that require memory only for features, rather than memory for feature-location bindings, is unclear.^9^ In both change detection, and the location-cued recall used to test memory in these experiments, disruption to feature-location bindings is sufficient to negatively impact memory performance, because this performance relies on recovering a feature given the location of a probe. However, if memory for features and memory for bindings are at least somewhat independent (e.g., Treisman & Zhang, 2006), performance in memory tasks that do not rely on binding information (e.g., free recall, or “global” recognition) may not be affected in the same way. Future work adapting the present design to use such tasks may shed light on precisely what component of memory is disrupted by novel bindings.

### Conclusion

These experiments show that external visual information is particularly disruptive to the contents of memory when it is novel. This is consistent with the assumptions of computational models of WM, which assume novelty-gated stimulus encoding, and suggests that this phenomenon may be consistent across WM for both verbal and visual materials.

## Electronic supplementary material

Below is the link to the electronic supplementary material.

(PDF 267 KB)

## Footnotes

1 Fiacconi and colleagues’ aim was not to assess effects of novelty on distraction, so this has no bearing on their conclusions; but it is relevant for interpreting their results insofar as my purpose here is concerned.

2 Note that with the Bayesian analysis methods used here, the emphasis on *a priori* choice of sample size that is important for frequentist methods (e.g., to prevent problems resulting from optional stopping) is unnecessary (e.g., Kass & Raftery, 1995; Kruschke & Liddell, 2018).

3 The unequal proportions resulted from a coding error that I did not discover until analyzing the data.

4 All data files and modeling and analysis scripts are available on the Open Science Framework at https://osf.io/wpyem/.

5 Following a reviewer’s suggestion, I also report results from a 2 (trial type: same-item vs. different-item) × 3 (probe type: positive vs. negative vs. intrusion) ANOVA.

6 I did not do the same with same-item positive trials, since they always involved presenting the target as the probe.

7 Namely, E3 reported by Fiacconi and Milliken (2012) does not show the superiority; E1 and E3 reported by Fiacconi and Milliken (2013) do, whereas E2 does not; and E1 and E3 reported by Cali et al., (2015) do.

8 Using the formula given by Cowan (2000), *k* = (*H* + *C**R* − 1)*N*—where *N* is the number of items in the memory array—the value would be 0.5*N* in both instances; and using the formula given by Pashler (1988), $k = N\frac {H-FA}{1-FA}$, it would be $\frac {5N}{6}$.

9 My thanks to a reviewer for highlighting this point.
