# Supplementary material for: Perceptual stimuli with novel bindings interfere with visual working memory
Source: Atten Percept Psychophys. 2021 Sep 3;83(8):3086–103. doi: 10.3758/s13414-021-02359-1 (PMC8550721; doi:10.3758/s13414-021-02359-1)
Supplement: Supplementary file 1 — (PDF 267 KB) [file 13414_2021_2359_MOESM1_ESM.pdf]

Novel bindings interfere with visual working memory: Supplemental material

Peter Shepherdson

University of Akureyri and University of Zurich

Novel bindings interfere with visual working memory: Supplemental material

Modelling methods

I fit the data with a modification of Bays, Catalao, and Husain's (2009) three-parameter mixture model. The parameters of this model can be described as p_u, the proportion of responses assumed to result from random guessing (i.e., drawn from a uniform distribution), p_n, the proportion of responses resulting from report of the wrong item (i.e., "swap" errors, drawn from a Von Mises distribution centred on a non-target item feature value), and κ, the precision of the information retained in memory (i.e., how narrow the distributions describing the memory items are). In addition, I included a fourth parameter to the model, p_p, the proportion of responses resulting from report of the change detection probe. The proportion of responses resulting from memory of the correct item, p_m, was thus, $p_m = 1 - p_u - p_n - p_p$. The overall response distributions were modelled as

$$(1 - p_g - p_s - p_p)\text{VM}(\mu_t, \kappa) + p_u\mathcal{U}(-\pi, \pi) + p_n\text{VM}(\mu_n, \kappa) + p_p\text{VM}(\mu_p, \kappa), \tag{1}$$

where $\text{VM}(\mu, \kappa)$ is a Von Mises distribution with mean μ and concentration parameter κ.

I modelled each participant's data separately, fitting them using maximum likelihood estimation and the *nlminb* function. All comparisons between parameter values across conditions were then performed using the same tests as I used for the raw data (see main manuscript).

Modelling results: Experiment 1

I conducted analyses on κ and p_m, reflecting the precision of responses and the probability they come from a distribution centred on the target value, respectively.

Does distractor novelty affect recall?

Different-item trials. Figure 1 displays condition means for κ (panel a) and p_m (panel b) for each probe type. One-way ANOVA provided weak evidence against an effect on κ ($BF = 0.58$), and for an effect on p_m ($BF = 1.31$). As is evident from the figure, in both cases the values were numerically higher for positive probe trials.

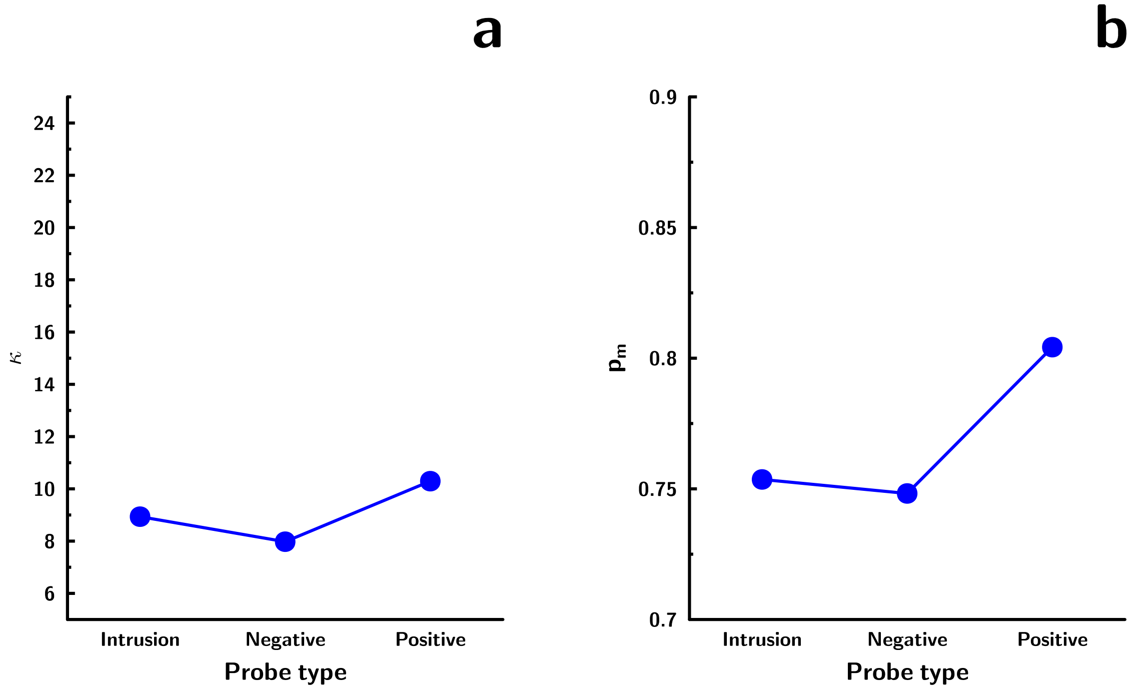

Figure 1. (a) Mean κ values obtained from fitting the four-parameter mixture model to data from different probe types in different-item trials from Experiment 1. (b) Mean p_m values.

Modelling results: Experiment 2

Does distractor novelty affect recall?

To provide for enough trials per condition for successful modelling, I combined data from CD+R and DJ+R trials when analysing effects of probe type. Figure 2 displays the

condition means for κ (panel a) and p_m (panel b) for each probe type.

Same-item trials. The mean value of κ was much higher for positive probe trials than for negative or intrusion probe trials, leading to a main effect of probe type ($BF = 1.78 \times 10^7$ relative to the intercept-only model). There was also evidence for an effect on p_m, with a lower mean value in positive than negative or intrusion probe trials ($BF = 13.74$ relative to the intercept-only model). However, examination of the values of the individual parameters influencing p_m suggested this was illusory. Namely, probe intrusions occurred with approximately 10% greater frequency in positive-probe trials than following the other two types of probes. Of course, since positive probes in same-item trials are identical to the target value, this means that this parameter and p_m trade off, so a better estimate of the "true" p_m value is obtained by adding the two together. After doing this, p_m ended up being substantially higher for positive probe trials (0.92) than for negative (0.80) or intrusion (0.78) probe trials.

Different-item trials. For different-item trials, there was weak evidence against a difference between the κ values of the different probe type trials ($BF = 0.38$ relative to the intercept-only model). However, p_m was higher in positive probe trials (.79) than in negative (.74) or intrusion (.73) probe trials, as indicated by a main effect of probe type ($BF = 23.37$ relative to the intercept-only model).

Distraction and location

To test a *post-hoc* hypothesis that same-item and different-item trials should reveal different patterns of error responses (see General Discussion), I also performed analyses on p_u (the probability of a response being drawn from a uniform distribution, a.k.a. "guessing"), p_n (the probability of a response being drawn from a distribution centred on the feature value of a non-target item, a.k.a. "swap errors"), and p_p (the probability of a response being drawn from a distribution centred on the feature value of the probe item from the change detection/direction judgement phase of a trial, a.k.a. "probe intrusions")

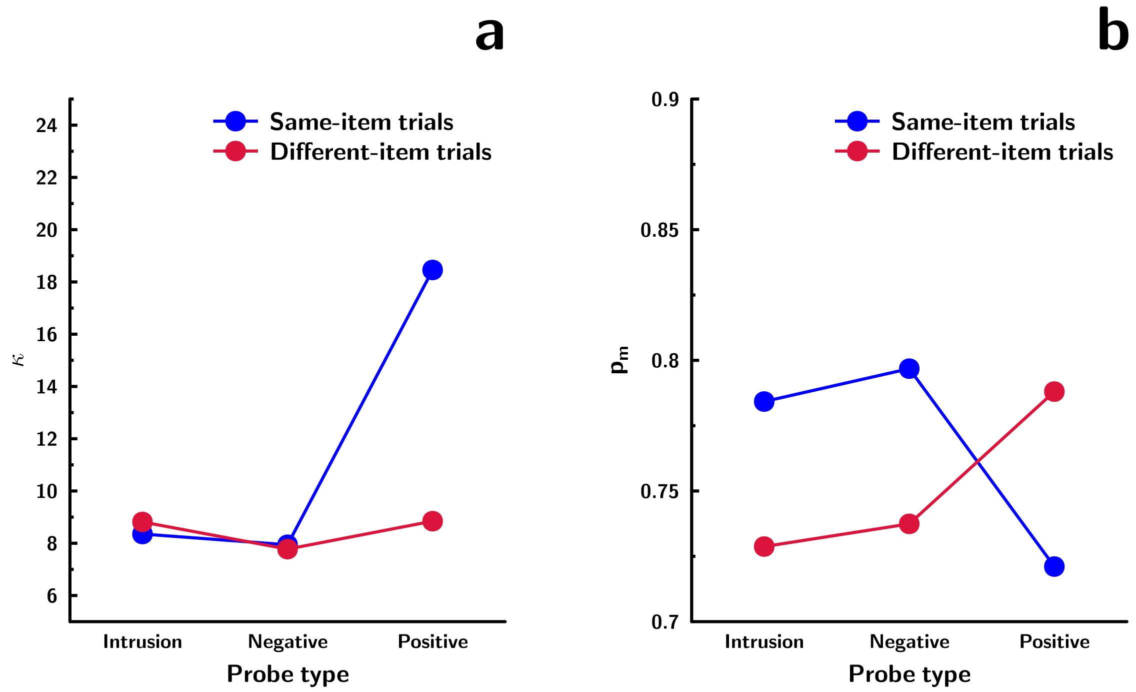

Figure 2. (a) Mean κ values obtained from fitting the four-parameter mixture model to data from different probe types in Experiment 2. (b) Mean p_m values.

on the combined CD+R and DJ+R data, split by probe type and trial type. Figure 3 displays condition means for each of these parameters. Bayesian ANOVA on each parameter showed that the best model in all three cases included a main effect of trial type. For p_u, the model containing only this effect was best ($BF = 4.92 \times 10^{18}$ relative to the intercept-only model), with higher values in different-item trials (.23) than in same-item trials (.07). For p_n, the best model contained main effects of both probe type and trial type ($BF = 5.23$ relative to the intercept-only model). However, this model was only very slightly superior to that with only a main effect of trial type ($BF = 1.08$). Numerically, the proportion of non-target responses was slightly higher for same-item trials (.03) than for different-item trials (.01). For p_p, the best model contained main effects of both probe type and trial type, and their interaction ($BF = 5.73 \times 10^{26}$ relative to the intercept-only model). The strong interaction is at least partly result of the trade-off

between probe-based and target-based responses for same-item positive probe trials, as mentioned in the previous subsection. Considering only negative and intrusion probe trials, there were more probe intrusions in same-item trials (.10) than in different-item trials (.01). Thus, as is evident from the figure, the direction of the trial type effects was notably opposed in the cases of p_u—which was higher for different-item trials—and p_p—which was higher for same-item trials. In other words, when participants erred in same-item trials, they tended to report the probe value with a relatively high frequency, whereas when they erred in different-item trials, they tended to more frequently guess (or at least, produce responses seemingly unrelated to the target, any of the non-targets, or the probe item).

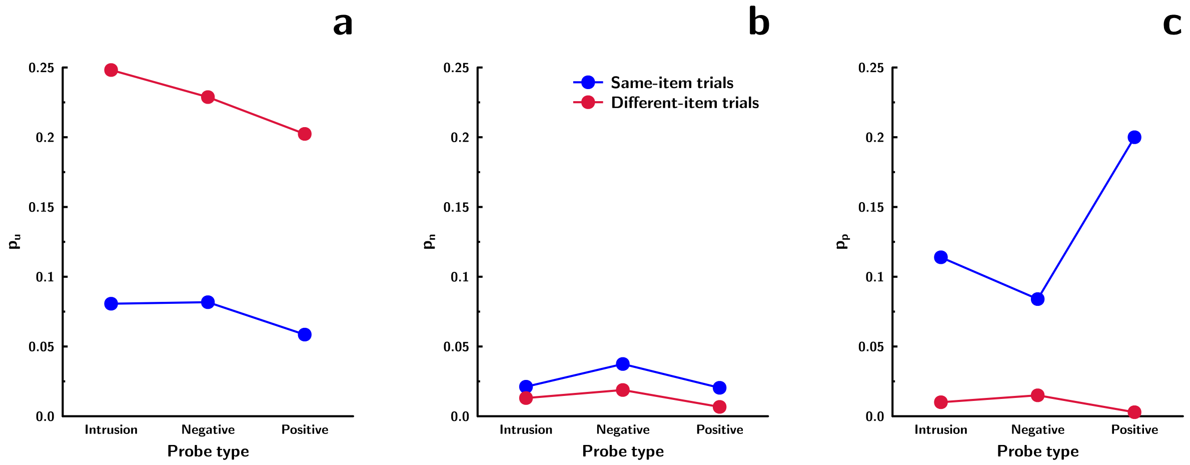

Figure 3. (a) Mean p_u values obtained from fitting the four-parameter mixture model to data from Experiment 2. (b) Mean p_n values. (c) Mean p_p values.

References

Bays, P. M., Catalao, R. F. G., & Husain, M. (2009). The precision of visual working memory is set by allocation of a shared resource. *Journal of Vision*, *9*, 1–11. doi: 10.1167/9.10.7.Introduction
